# A YAP/TAZ–ARHGAP29–RhoA Signaling Axis Regulates Podocyte Protrusions and Integrin Adhesions

**DOI:** 10.3390/cells12131795

**Published:** 2023-07-06

**Authors:** Manuel Rogg, Jasmin I. Maier, Martin Helmstädter, Alena Sammarco, Felix Kliewe, Oliver Kretz, Lisa Weißer, Clara Van Wymersch, Karla Findeisen, Anna L. Koessinger, Olga Tsoy, Jan Baumbach, Markus Grabbert, Martin Werner, Tobias B. Huber, Nicole Endlich, Oliver Schilling, Christoph Schell

**Affiliations:** 1 Institute of Surgical Pathology, Medical Center, Faculty of Medicine, University of Freiburg, 79106 Freiburg, Germany; 2 Department of Medicine IV, Medical Center, Faculty of Medicine, University of Freiburg, 79106 Freiburg, Germany; 3 Department of Anatomy and Cell Biology, University Medicine Greifswald, 17489 Greifswald, Germanynicole.endlich@uni-greifswald.de (N.E.); 4 III. Department of Medicine, University Medical Center Hamburg-Eppendorf, 20251 Hamburg, Germany; 5 Hamburg Center for Kidney Health (HCKH), University Medical Center Hamburg-Eppendorf, 20251 Hamburg, Germany; 6 Institute for Computational Systems Biology, University of Hamburg, 22607 Hamburg, Germany; olga.tsoy@uni-hamburg.de (O.T.);; 7 Department of Mathematics and Computer Science, University of Southern Denmark, 5230 Odense, Denmark; 8 Department of Urology, Medical Center, Faculty of Medicine, University of Freiburg, 79106 Freiburg, Germany; 9 Freiburg Institute for Advanced Studies (FRIAS), University of Freiburg, 79106 Freiburg, Germany

**Keywords:** podocyte, glomerular kidney disease, EPB41L5, Yurt, YAP/TAZ, ARHGAP29, mechanotransduction

## Abstract

Glomerular disease due to podocyte malfunction is a major factor in the pathogenesis of chronic kidney disease. Identification of podocyte-specific signaling pathways is therefore a prerequisite to characterizing relevant disease pathways and developing novel treatment approaches. Here, we employed loss of function studies for *EPB41L5* (*Yurt*) as a central podocyte gene to generate a cell type-specific disease model. Loss of *Yurt* in fly nephrocytes caused protein uptake and slit diaphragm defects. Transcriptomic and proteomic analysis of human *EPB41L5* knockout podocytes demonstrated impaired mechanotransduction via the YAP/TAZ signaling pathway. Further analysis of specific inhibition of the YAP/TAZ-TEAD transcription factor complex by *TEADi* led to the identification of *ARGHAP29* as an *EPB41L5* and YAP/TAZ-dependently expressed podocyte RhoGAP. Knockdown of *ARHGAP29* caused increased RhoA activation, defective lamellipodia formation, and increased maturation of integrin adhesion complexes, explaining similar phenotypes caused by loss of *EPB41L5* and *TEADi* expression in podocytes. Detection of increased levels of ARHGAP29 in early disease stages of human glomerular disease implies a novel negative feedback loop for mechanotransductive RhoA—YAP/TAZ signaling in podocyte physiology and disease.

## 1. Introduction

Glomerular disease and related podocytopathies are central factors in the pathogenesis of chronic kidney disease (CKD) [1,2]. Uncovering novel disease mechanisms in podocytopathies is therefore an unmet medical need in light of the increasing global disease burden due to CKD [3]. Podocytes are specialized epithelial cells that, together with endothelial cells and the glomerular basement membrane (GBM), form the kidney filtration barrier. One exceptional feature of podocytes is their highly complex cellular morphology, characterized by an arborized network of interdigitating cell protrusions enwrapping glomerular capillaries [4]. Any kind of injury results in a dramatic reconfiguration of podocyte morphology, highlighted by the simplification (effacement) of podocyte foot processes (FP), finally translating into progressive podocyte detachment [4]. Based on these observations, it is well established that podocytes rely on specific cell-matrix adhesions and an elaborate actin cytoskeleton machinery to maintain anchorage towards the GBM [5,6]. However, underlying signaling networks involving small RhoGTPases (e.g., RhoA, Rac1, CDC42) are still incompletely understood [7,8,9].

Aside from the complex RhoGTPase signaling network, integrin adhesion complexes (IACs; focal adhesions) not only establish physical linkage between the actin cytoskeleton and the extracellular matrix (ECM) but also serve as integrative signaling hubs (e.g., mediating substrate sensing, ECM remodeling, and integration of mechanical cues) [10,11]. Given their central role in fine-tuning mechanotransduction, IACs are essentially required to withstand glomerular filtration forces and ultimately prevent podocyte detachment [12,13,14,15]. Moreover, a series of recent studies have highlighted the cell type-specific composition of these molecular machineries [2,5,8,12,16,17]. The relevance of these subcellular complexes is demonstrated when podocytes are challenged due to increased extracellular and intracellular mechanical stressors, translating into adaptive remodeling of the ECM-IAC-actin cytoskeleton axis [18,19]. In this context, we have previously identified EPB41L5 as a podocyte-enriched IAC component mediating a mechanotransduction cascade [17,20,21,22]. It has been previously shown that EPB41L5 is a podocyte-specific regulator of IACs, cell polarity, and actomyosin contractility [17,20,21]. Loss of *Epb41l5* results in foot process effacement and podocyte cell detachment. Furthermore, it affects GBM development and presents with the development of nephrotic syndrome [17,22,23]. Mechanistically, the absence of *EPB41L5* impairs mechanotransduction via IACs by decreasing ARHGEF18–RhoA–actomyosin signaling and by reducing the recruitment of adaptive IAC proteins such as ACTN4 and PDLIM5. In general, mechanotransduction is implemented by several signaling processes, including RhoGTPase signaling via the RhoA-ROCK-actomyosin pathway, transcriptional regulation by nuclear translocation of transcription factors (e.g., YAP/TAZ coactivators), force-mediated spatial reorganization of chromosomes, and force-induced protein conformational changes (e.g., of the IAC component Talin-1) [11,24,25,26]. Previous studies have demonstrated that activation and loss of RhoA, Talin-1 (TLN1), and YAP/TAZ signaling are associated with podocytopathy [15,27,28,29,30,31,32].

Given the versatile role of mechanosignaling in podocytes, we hypothesized that podocytes rely on a cell type specific molecular repertoire to modulate mechanotransduction in health and disease. Therefore, we asked if mechanotransduction alters the expression of podocyte specific regulatory genes and if these genes are functionally involved in regulation of IACs and cell protrusions? In this study, we focused on *EPB41L5* and YAP/TAZ signaling as podocyte specific disease proxy to identify molecular consequences of altered mechanotransduction in podocytes. Here, we describe underlying mechanisms and consequences of these alterations and identify novel regulatory pathways for mechanotransductive signaling in podocyte disease.

## 2. Methods

### 2.1. Nephrocyte Analysis

*D. melanogaster* stocks were cultured on standard cornmeal molasses agar food and maintained at 25 °C. The *Drosophila* orthologue (CG 9764 *Yurt*) of mammalian EPB41L5 was identified using the DIOPT DRSC Integrative Ortholog Prediction Tool (Harvard Medical School). Virgins of prospero-Gal4 (gift from Barry Denholm) were crossed to UAS-Yurt-RNAi males (VDRC TID 28674GD) for a GCN-specific knockdown of Yurt.

For RNAi-based functional analysis of *Drosophila* Garland nephrocytes, virgins from the MHC-ANF-RFP, HandGFP, and Dot-Gal4 (gift from Zhe Han) transgenic lines were crossed to UAS-Yurt-RNAi (VDRC TID 28674GD) males at 25 °C. 2 days after crossing, flies were transferred to small collection cages with grape juice agar plates to collect the embryos for 24 h at 25 °C. Collected embryos were aged for 48 h at 29 °C and then subjected to examination of the RFP accumulation in pericardial nephrocytes under fluorescent microscopy. The RFP mean fluorescence intensity of GFP-positive areas was measured to quantify the uptake efficiency.

For transmission electron microscopy, *Drosophila* Garland cells, including the stomach, were removed and immersion fixed in 4% PFA and 1% glutaraldehyde (Roth, Germany; in phosphate buffer, overnight at 4 °C). After fixation, samples were embedded in a drop of low-melting agarose and contrasted with 0.5% OsO4 (Roth, Germany; 1 h at room temperature). Finally, the samples were dehydrated in an ascending ethanol series with propylene oxide. Samples were embedded in epoxy resin (Durcopan, Plano, Wetzlar, Germany). Ultrathin sections (40 nm) were cut using an UC6 ultramicrotome (Leica, Germany) and analyzed using a Philipps CM 100 transmission electron microscope. For quantification, the Olympus ITEM software was used.

### 2.2. Cell Culture

Human immortalized podocytes (AB8/13) were obtained from Moin A. Saleem (Bristol University, UK). Podocyte culture medium was composed of RPMI-1640 GlutaMAX (Thermo Fisher Scientific Inc., Waltham, MA, USA, #61870036) supplemented with 10% FCS (Merck KGaA, Darmstadt, Germany, #S0615), 10 µg/mL ITS (Merck, #11074547001), 1:1000 NEAA (Thermo Fisher Scientific, #11140050), 5 mM HEPES (Thermo Fisher Scientific, #15630-056), 100 µM Sodium-Pyruvate (Thermo Fisher Scientific, #11360-039), and penicillin-streptomycin (Thermo Fisher Scientific, #15140122). Cell culture, differentiation, and phenotypic characterization of CRISPR/Cas9 generated *EPB41L5* knockout (KO) and wild-type (WT) podocytes were previously described [17,22]. The WT-1 control represents the parental podocyte cell line transfected by an empty vector control. A non-mutated WT-2 control was generated in parallel to KO clones arising from single cell clones. If not stated otherwise, cells were analyzed in non-confluent culture conditions in all experiments. Before seeding into experiments, cells were differentiated and continuously cultured below 70% confluence. Podocyte cell lines expressing *ARHGAP29* shRNAs, *ARHGAP29*, *TEADi,* and respective control plasmids were generated via transduction with respective lentiviral particles produced in HEK293T/17 cells (ATCC, Manassas, VA, USA, CRL-11268). Before experimental analysis, *TEADi* and *EGFP* control cells were induced with doxycycline (2 µg/mL) for 24 h. HEK293T/17 cells were cultured in DMEM GlutaMAX (Thermo Fisher Scientific, #31966-021) supplemented with 10% FCS. Routine testing of cell lines for contamination with Mycoplasma was performed using a commercial PCR kit (Mycoplasma PCR detection kit, Hiss Diagnostics GmbH, Freiburg, Germany).

### 2.3. Expression Plasmids

Human *ARHGAP29* (NM_004815.4) cloned into the pGenLenti1200 plasmid was purchased (GeneScript, OHu13454C). Luciferase was cloned into pWPXLd; pWPXLd was a gift from Didier Trono (Addgene plasmid # 12258; http://n2t.net/addgene:12258 (last accessed 3 May 2023); RRID: Addgene_12258). Previously validated shRNAs targeting ARHGAP29 (shRNA-1: CCTTAAGTTCCAACTCTATTT, shRNA-2: TAAGATAGGTGGATTCGTATT) cloned into pLKO.1 plasmids were purchased (Merck, Germany, TRCN0000426540 and TRCN0000422937) [33]. Scramble pLKO.1 shRNA “control-1” (shCtrl.-1) was a gift from David Sabatini (Addgene plasmid #1864; http://n2t.net/addgene:1864 (last accessed 3 May 2023); RRID:Addgene_1864) and “control-2” (shCtrl.-2) from William Hahn (Addgene plasmid #42559; http://n2t.net/addgene:42559 (last accessed 3 May 2023); RRID:Addgene_42559). The tetracycline-inducible pInducer20 EGFP-TEADi plasmid inhibiting the interaction of YAP1 and TAZ with TEAD transcription factors was a gift from Ramiro Iglesias-Bartolome (Addgene plasmid #140145; http://n2t.net/addgene:140145 (last accessed 3 May 2023); RRID:Addgene_140145) [34]. A lentiviral Tetracycline-inducible EGFP expression plasmid was cloned and used as a negative control for the pInducer20 EGFP-TEADi plasmide.

### 2.4. Antibodies

The following antibodies and dyes were applied for immunofluorescence (IF) and western blot (WB) analysis: NPHS1 (GP-N2, PROGEN, Heidelberg, Germany, IF 1:300), EPB41L5 (HPA037564, Atlas Antibody, Bromma, Sweden, IF 1:200), YAP1 (14074, Cell Signaling, Danvers, MA, USA, IF 1:200, WB 1:1000), YAP/TAZ (8418, Cell Signaling, IF 1:200, WB 1:1000), ARHGAP29 (sc-365554, Santa Cruz, Dallas, TX, USA, IF 1:50, WB 1:500), EPB41L5 (HPA037563, Atlas Antibody, Bromma, Sweden, WB 1:1000), TUBA (T9026, Merck, Darmstadt, Germany, WB 1:3000), RhoA (2117, Cell Signaling, WB 1:1000), PXN (610051, BD Biosciences, Franklin Lakes, NJ, USA, IF 1:300), Luciferase (NB100-1677, Novus Biological, Bio-Techne GmbH, Wiesbaden, Germany, WB 1:1000), Hoechst 33342, trihydrochloride, trihydrate (H3570, Thermo Fisher Scientific, Waltham, MA, USA, IF 1:1000) Alexa Fluor phalloidin 488 and 555 (A12379, A30106, Thermo Fisher Scientific, IF 1:500). Wheat Germ Agglutinin (WGA)-FITC (FL-1021-5, Vector Laboratories, Newark, CA, USA 1:1000). For immunofluorescence fluorophore conjugated secondary antibodies (A-31572, A-21127, A31570, and A-21450, Thermo Fisher Scientific, IF 1:500) and for western blot HRP-linked, anti-mouse (P0447, Dako, Agilent, Sanata Clara, CA, USA, WB 1:10,000), anti-goat (P0449, Dako, WB 1:5000), or anti-rabbit (7074, Cell Signaling, WB 1:1000) antibodies were applied.

### 2.5. Western Blot Analysis

For equalized cell densities in western blot analysis, cells were detached using trypsin, and 1 million cells per condition were seeded into a new 10 cm cell culture dish and cultured for another 24 h at 37 °C. Subsequent cell lysis in RIPA buffer, SDS-polyacrylamide gel electrophoresis (SDS-PAGE), western blotting, and immunodetection using the HRP-ECL detection reaction were performed as previously described [35]. Cell lysates were equalized based on measurement of protein concentration (BCA protein assay kit, #23225, Thermo Fisher Scientific), and equal amounts of protein were loaded for western blot analysis. Fiji ImageJ v1.52 was used for densitometric analysis of western blot bands. Band densitometries were normalized to TUBA and presented as relative values to the mean of control samples. If possible, all independent experimental replicates used for statistical analysis were measured in one western blot experiment. For analysis of ARHGAP29 expression in *EPB41L5* podocytes, independent experiments were normalized to the mean ARHGAP29 expression of wild-type samples, and these relative values were pooled for statistical analysis and graphical presentation.

### 2.6. Analysis of Active RhoA

Levels of active (GTP bound) RhoA were determined by applying an ELISA-based assay kit (G-LISA, BK124; Cytoskeleton Inc., Denver, CO, USA). Cell density was equalized as described for western blot analysis. Subsequently, podocytes were serum starved for 24 h and processed for cell lysis. Lysates were equalized according to protein concentration and further processed for G-LISA analysis according to the manufacturer’s instructions.

### 2.7. Immunofluorescence, Cell Spreading, and Integrin Adhesion Complex Analysis

Immunofluorescence (IF), initial cell spreading, and integrin adhesion complex (IAC) analysis of podocytes was performed as previously described in detail [12,35]. Podocytes were cultured on collagen IV (Merck, #C5533) coated 8-well chamber slides (Ibidi GmbH, Gräfelfing, Germany, #80827) for IAC and IF analysis or collagen IV pre-coated 8-well chamber slides (Ibidi GmbH, #80822) for cell spreading assays. For spreading analysis of *EPB41L5* WT and KO podocytes, glass coverslips were coated with 50 µg/mL laminin (L2020, Merck), fibronectin (354008, Corning, NY, USA), collagen IV (C5533, Merck), or vitronectin (SRP3186, Merck). For analysis of IACs and cell morphology, podocytes were cultured in a 37 °C cell culture incubator for 24 h or for initial cell spreading analysis for 30 min after seeding. IF staining of the IAC component Paxillin (PXN), of F-Actin by fluorophore-labeled phalloidin, and of cell nuclei by Hoechst was performed. Cells were imaged, and individual cells and IACs were morphologically analyzed using the Fiji ImageJ v1.52 software. For initial cell spreading experiments, at least 100 cells and, for IAC analysis, 25 cells per genotype were analyzed for each experiment. For analysis of nuclear YAP or YAP/TAZ translocation, cells were cultured on collagen IV-coated 8-well chamber slides and analyzed, respectively, 4 h or 3 days after seeding. Cell nuclei of individual cells were segmented based on Hoechst staining, and nuclear mean fluorescence intensity (MFI) was measured using QuPath (v0.3.2) [36]. For each experimental replicate, at least 120 cells per genotype were analyzed. For analysis at 4 h, nuclear YAP MFI was normalized to Hoechst MFI to correct for differences in nuclear flattening at these time points after seeding. For analysis of cell size and lamellipodia type, cells were processed as for IAC analysis, and at least 100 cells per genotype for each experiment were analyzed. For lamellipodia analysis, lamellipodia were classified as usual type (one dominant and coherent lamellipodium), atypical (fragmented, multi- or pseudopod-like) or minimally formed. An AxioObserver microscope together with the ZEN 3.2 software package was used for IF analysis (Carl Zeiss AG, Oberkochen, Germany). The microscope was equipped with a Colibri 7 light source, an Axiocam 702 monochrome camera, and an ApoTome.2 device and Zeiss fluorescence filter sets: 49 DAPI, 38 GFP, 43 HE dsRed, and 50 Cy5.

### 2.8. Glomerular Disease Analysis

Analysis of human kidney samples (male patients, age 52 to 72 years old, nephrectomy samples, impaired renal function (mean eGFR < 45 mL/min), and established diagnosis of arterial hypertension) was approved by the ethic board of the university medical center Freiburg (EK 21/1288; 18/512). Processing and IF staining of human formalin-fixed paraffin embedded (FFPE) tissue were previously described in detail [12]. In brief, 2 µm sections of FFPE tissue were subjected to heat-induced antigen retrieval (HIAR) in ph6 citrate buffer. Subsequently, sample blocking (5% BSA in PBS) and indirect IF staining were performed. Primary antibodies were applied for 20 h at 4 °C and secondary fluorophore-tagged antibodies for 45 min at room temperature. Antibodies and fluorescent dyes were diluted in blocking solutions as described above. Whole slide scanning of kidney sections was performed using an Akoya Fusion microscope (Akoya Biosciences Inc., Marlborough, MA, USA). Images were processed (demultiplexing of fluorescence dyes and background subtraction) using the InForm v2.6 software (Akoya Biosciences) and analyzed using the QuPath v0.3.2 software [36]. For image analysis, glomeruli were classified as not/very mildly damaged (control group), moderately damaged (damaged group), or sclerosed (excluded from analysis) based on the structural integrity of SD and FP architecture and of the overall glomerular structure (evaluated by WGA, NPHS1, and Hoechst staining). Selected glomeruli were analyzed for mean ARHGAP29 IF staining intensity within the podocyte compartment by segmentation of the NPHS1 positive region per glomerulus. Control and damaged glomeruli were matched per sample for statistical analysis to adjust for differences in overall fluorescence intensities between individual samples. At least 20 glomeruli per sample were analyzed. mRNA expression analysis of human glomeruli and respective statistical analysis were retrieved from https://www.nephroseq.org (accessed on 28 November 2022).

### 2.9. GlomAssay

Glomeruli of nephrin::CFP mice were isolated with magnetic dynabeads as previously described [37]. Afterwards, isolated glomeruli were cultured on collagen IV-coated μ-slides (ibidi GmbH, Munich, Germany) in phenol red-free RPMI 1640 medium (Lonza Group Ltd., Basel, Switzerland) supplemented with 10% FBS (Thermo Fisher Scientific), 100 U/mL penicillin, and 0.1 mg/mL streptomycin (Life technologies). Glomeruli were treated with Doxorubicin (50 µM, Sigma-Aldrich, St. Louis, MI, USA). After 3 days, RNA samples from Doxorubicin-treated and untreated control glomeruli were prepared for sequencing on a 5500xl SOLiD™ system (Life Technologies, Carlsbad, CA, USA) using recommended protocols as described previously [38]. Subsequently, the samples were normalized using DeSeq2 (Bioconductor). For the evaluation of statistical significance, a q-value was calculated using the Wald test, followed by a Benjamini-Hochberg multiple test correction. The result was considered statistically significant when the q value was below 0.05.

### 2.10. RNA Sequencing

To equalize cell density for transcriptome analysis, cells were detached using trypsin, and 1 million cells per condition were seeded into a new 10 cm cell culture dish and cultured for another 24 h at 37 °C. Subsequently, cells were washed in PBS and harvested by scraping. The Monarch Total RNA Miniprep kit (T2010S, New England Biolabs Inc., Ipswich, MA, USA) was applied for RNA isolation according to the suppliers protocol. Sample quality was determined using an Agilent Fragment Analyzer (RQN 10 for all samples). Poly(A)mRNA selection, library preparation, Illumina 2 × 150 bp paired-end sequencing on an NovaSeq 6000 system and demultiplexing with adapter trimming (bcl2fastq v2.19) was performed by the GENEWIZ Germany GmbH. For comparison of *EPB41L5* WT and KO podocytes (two independent replicates, total of eight samples), the NEBNext Ultra II Directional RNA Library Prep kit was applied. For analysis of *TEADi* and *EGFP* (control) podocytes (three independent replicates, total of six samples; doxycycline induced for 24 h), the NEBNext Ultra II RNA Library Prep kit (New England Biolabs) was used. The Galaxy Europe bioinformatics platform was used for further data processing as previously described [35]. Finally, DESeq2 was applied for statistical analysis of differential gene expression. The GenePattern software was used for gene set enrichment analysis (GSEA) as previously described [35]. Transcription factor enrichment analysis (TFEA) was performed using ChEA3 [39]. GSEA and TFEA details and results can be found in supplementary Tables S1 and S4. In vivo transcriptome analysis (for correlation procedures) of murine podocytes for genes expressed or specific enriched in podocytes was previously described [8]. Consensus lists of the human matrisome and integrin adhesome were applied as previously described (Supplementary Table S3) [17].

Sequencing data have been deposited in the NCBI Gene Expression Omnibus and are accessible via GEO series accession numbers GSE220299 and GSE220221 (differential expression analysis is enclosed in Supplementary Tables S1 and S4).

### 2.11. Proteomics

**Figure 1:**
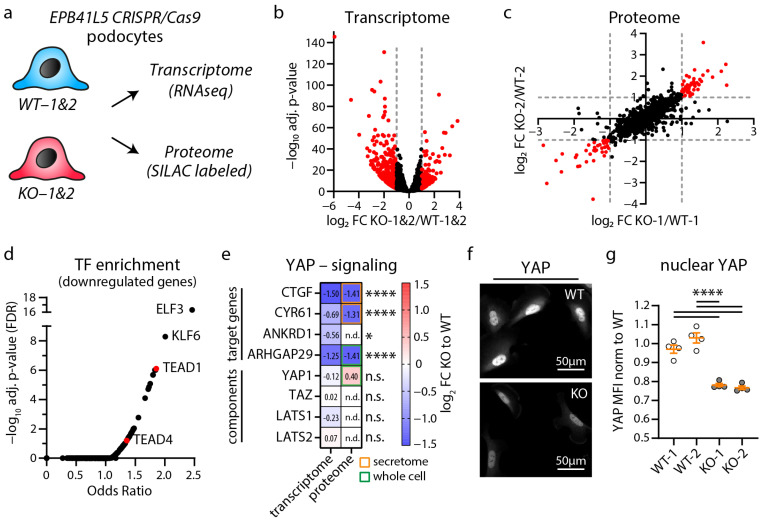
Transcriptome and proteome analysis of *EPB41L5* knockout podocytes reveals impaired YAP/TAZ signaling. (**a**) Schematic depicting transcriptome and proteome analysis of CRISPR/Cas9 generated *EPB41L5* knockout (KO) and wildtype (WT) podocytes. (**b**) Volcano plot of RNA sequencing (transcriptome) analysis of two WT and KO cell lines indicate significant regulation of 595 gene transcripts (red dots). Significance (red dots) was defined as log_2_ fold change (FC) > 1 or <−1 and adjusted *p*-value < 0.05. Two replicates per cell line were analyzed. (**c**) Scatter plot of SILAC based quantitative LC-MS (proteome) analysis of whole cell lysates of *EPB41L5* WT and KO podocytes indicates significant regulation of 84 proteins (red dots). Significance was defined as log_2_ fold change (FC) > 1 or <−1 in both SILAC pairs of WT and KO cells (KO-1 to WT-1 and KO-2 to WT-2). (**d**) Enrichment analysis of transcription factors (TF) for significantly downregulated transcripts in KO podocytes. Red dots indicate enrichment of gene transcripts regulated by YAP/TAZ-TEAD (TEAD1 and TEAD4) mediated transcription signaling. (**e**) Transcriptome and proteome analysis of *EPB41L5* KO podocytes shows reduced expression levels of YAP target genes (secretome—data derived from a previously published proteome analysis of secreted proteins [22]). (**f**,**g**) Immunofluorescence analysis of nuclear localization of YAP confirms reduced YAP levels in *EPB41L5* KO podocytes. Scatter plot dots indicate mean nuclear fluorescence intensity (MFI) of 4 experiments as used for statistical analysis (white dots indicate WT and grey dots KO cells); error bars indicate mean and S.E.M.; * *p* < 0.01; **** *p* < 0.0001; n.s.—not significant; n.d.—not detected.

Quantitative MS analysis of WT and *EPB41L5* KO podocytes was performed employing stable isotope labeling by amino acids in cell culture (SILAC) for 14 days. Preparation and LC-MS/MS analysis of SILAC labeled cells were performed at the core facility for proteomics at the University of Freiburg as described before [22,40]. Log2 fold changes were calculated for each pair of WT and KO podocytes using MaxQuant, and log2 fold changes of detected proteins were used for subsequent analysis. See Supplementary Table S2 for proteome analysis. Previous published secretome and adhesome analysis of WT and *EPB41L5* KO podocytes was used for correlative analysis was previously published, as indicated in Figure legends (Figure 1e and Figure S1f) [17,22].

The LC-MS/MS data was uploaded to the MassIVE repository (part of the ProteomeXchange consortium). Data can be accessed using the MassIVE accession number MSV000091196.

### 2.12. Statistical Analysis

**Figure 2:**
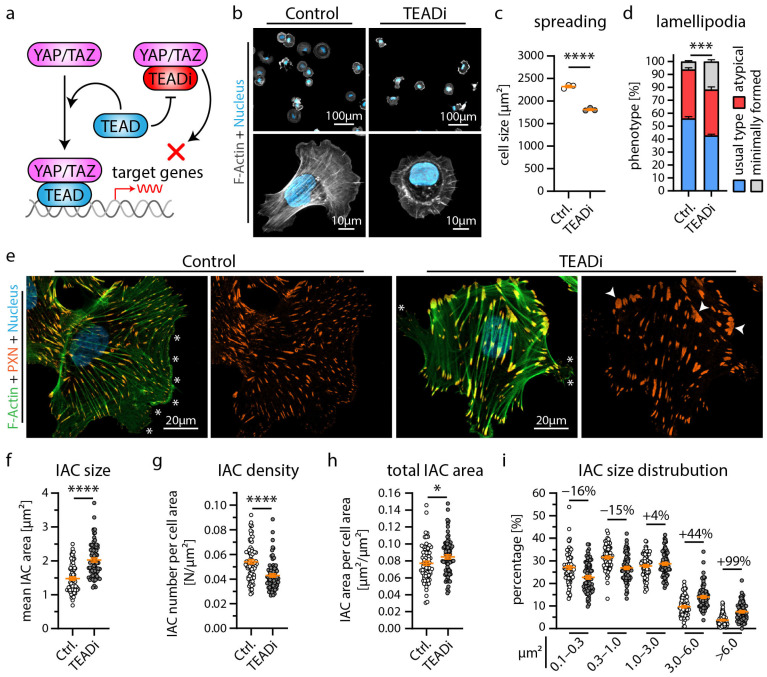
Inhibition of YAP/TAZ-TEAD mediated transcription alters cytoskeletal function of podocytes. (**a**) Schematic depicting the specific inhibition of YAP/TAZ-TEAD complex formation by expression of the YAP/TAZ binding domain of TEAD transcription factors (TEADi system) [34]. (**b**,**c**) Cell spreading analysis of control vector and *TEADi* podocytes shows impaired initial cell spreading (after 30 min) by *TEADi* expression on collagen IV. Cells were stained with Phalloidin (F-Actin) and Hoechst (cell nucleus). Scatter plot dots indicate the mean cell size of 3 individual experiments used for statistical analysis (white dots indicate Ctrl. and grey dots *TEADi* cells). (**d**,**e**) Immunofluorescence analysis of full spread cells (24 h) shows increased numbers of cells with atypical (fragmented pseudopod-like) or minimally formed lamellipodia (white asterisks) as a result of *TEADi* induction. Percentage of cells exhibiting regular formed lamellipodia (usual type) was used for statistical comparison (percentages of 3 experiments were used for statistical analysis). (**e**) Immunofluorescence staining of the integrin adhesion complex (IAC) component Paxillin (PXN), F-Actin (Phalloidin) and of cell nuclei (Hoechst) was performed. Lamellipodia (white asterisks) of *TEADi* cells appeared less coherent. Moreover, an increased number of large IACs was observed (white arrowheads). (**f**–**i**) Quantitative analysis of IAC morphology confirms increased IAC size and increased appearance of larger IACs. Scatter plot dots indicate mean values of individual cells used for statistical analysis; 3 independent experiments with 25 cells per experiment and genotype (total of 75 cells per genotype) were analyzed. Percentages indicate relative change to control for respective IAC size classes. Error bars indicate mean and S.E.M.; * *p* < 0.05; *** *p* < 0.001; **** *p* < 0.0001.

**Figure 3:**
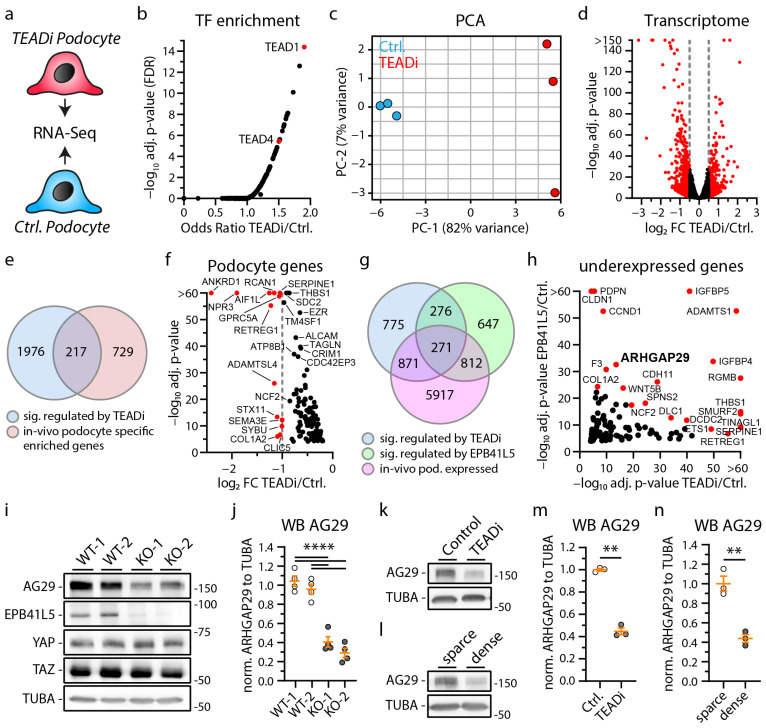
The YAP/TAZ-TEAD complex controls expression of podocyte specific genes like ARHGAP29. (**a**) Schematic illustrating the transcriptome analysis of *TEADi* and control (*EGFP*) expressing podocytes. (**b**) Transcription factor (TF) enrichment analysis for significant regulated genes transcripts confirms enrichment of TEAD1 and TEAD4 target genes as a consequence of expression of *TEADi*. (**c**) Principal component analysis (PCA) demonstrates clear transcriptional separation of control and *TEADi* podocytes. (**d**) Volcano plot shows significant alteration of 1274 gene transcripts (red dots). Significance for this plot was defined as log_2_ fold change (FC) > 0.5 or <−0.5 and adjusted *p*-value < 0.05. (**e**) Venn diagram depicts overlap of 217 podocyte genes transcripts that are significantly regulated by *TEADi* expression (*p*-value < 0.0001) and that are highly podocyte specific enriched in vivo [8]. (**f**) Volcano plot of these genes reveals strongly TEAD TF dependent expression of many genes with essential functions in podocytes (only genes with reduced expression followed by *TEADi* expression are shown, red dots indicate genes with a log_2_ fold change <−1). (**g**) Venn diagram depicts overlap of 271 podocyte gene transcripts that are significantly regulated by *TEADi* expression (*p*-value < 0.0001) and by loss of *EPB41L5* (*p*-value < 0.0001) and that are expressed in podocytes in vitro. [8] (**h**) Volcano plot shows 118 of these 271 genes that are less expressed in consequence of TEADi expression and loss of *EPB41L5* (red dots indicate gene transcripts with highly significant adjusted *p*-values). (**i**–**n**) Western blot confirms reduced expression of ARHGAP29 in *EPB41L5* KO cells, *TEADi* expressing cells and as a result of confluent (dense) cell culture conditions (3 or 4 independent replicates per experiment were used for statistical analysis as indicated by scatter plot dots; white and gray dots indicate experimental conditions). Alpha-Tubulin (TUBA) was used as loading control and quantification is presented relative to mean expression of control cells. Error bars indicate mean and S.E.M.; ** *p* < 0.01; **** *p* < 0.0001.

**Figure 4:**
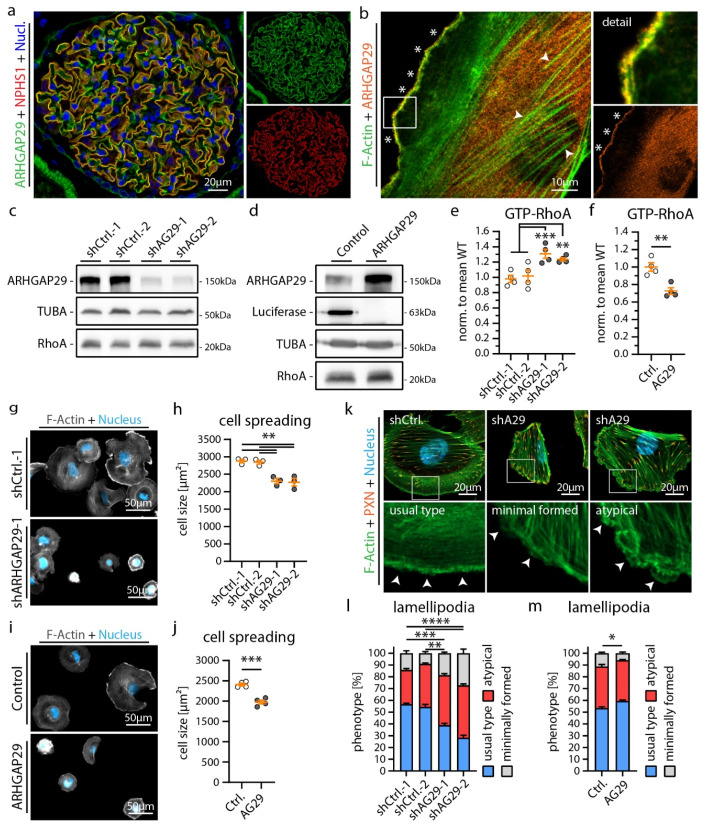
ARHGAP29 is required for generation of lamellipodia in podocytes. (**a**) Immunofluorescence analysis of human glomeruli confirms podocyte specific enrichment of ARHGAP29 (green). NPHS1 (red) was used to label the podocyte compartment and Hoechst to stain cell nuclei (blue). (**b**) Immunofluorescence analysis of cultured human podocytes reveals subcellular localization of ARHGAP29 to the leading edge of lamellipodia (white asterisks) and diffuse positivity of the cytosol but not of F-Actin (white arrowheads). F-Actin (Phalloidin) staining was applied to label actin fibers and lamellipodia. (**c**,**d**) Western blot analysis of ARHGAP29 demonstrates successful generation of shRNA mediated *ARHGAP29 (AG29)* KD or *ARHGAP29* overexpressing podocytes. Non-targeting shRNAs or Luciferase were expressed as negative controls. Alpha-Tubulin (TUBA) was used as loading control. No marked alteration of RhoA was detected. (**e**,**f**) Active GTP-bound RhoA analysis confirms RhoGAP function of ARHGAP29 for RhoA in podocytes. Scatter plot dots indicate independent replicates used for statistical analysis. shCtrl.−1 & −2 were pooled for statistical analysis (white dots indicate Ctrl. and grey dots KD or overexpression cells). (**g**–**j**) Knockdown and overexpression of *ARHGAP29* impairs initial cell spreading (30 min) on collagen IV. Cells were stained by Phalloidin (F-Actin) and Hoechst (cell nucleus). Scatter plot dots indicate mean cell size of 3 (**h**) or 4 (**j**) experiments used for statistical analysis. (**k**–**m**) Immunofluorescence analysis of full spread cells (24 h) reveals increased numbers of cells with atypical (fragmented pseudopod-like) or minimally formed lamellipodia as a result of *ARHGAP29* KD. Representative images show representative cells for different classes of lamellipodia (white arrowheads). Percentage of cells exhibiting regular formed lamellipodia (usual type) was used for statistical comparison (3 experiments were analyzed and percentages per experiment and condition were used for statistical analysis). Immunofluorescence staining of the IAC component Paxillin (PXN), F-Actin (Phalloidin) and of cell nuclei (Hoechst) was performed. Error bars indicate mean and S.E.M.; * *p* < 0.05; ** *p* < 0.01; *** *p* < 0.001; **** *p* < 0.0001.

**Figure 5:**
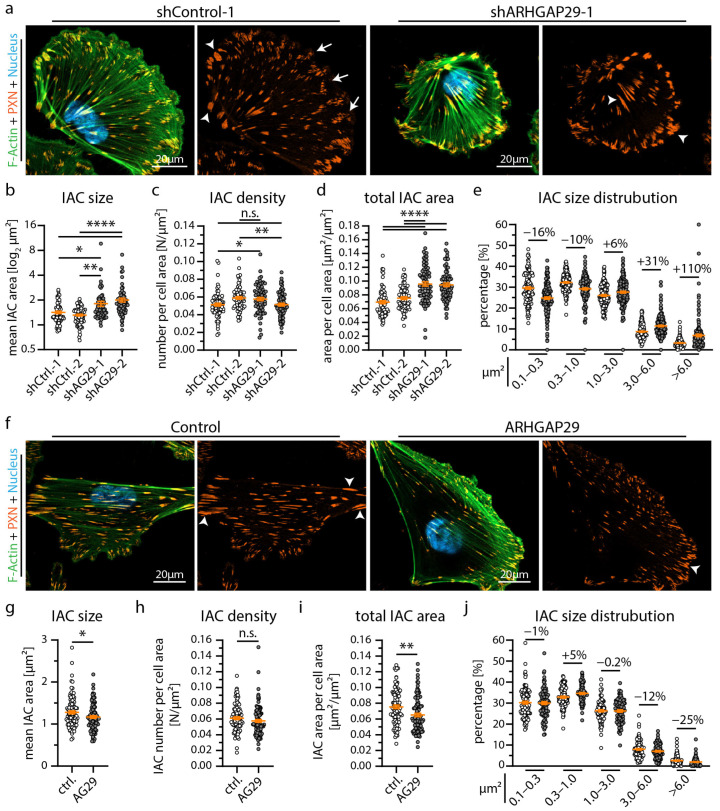
ARHGAP29 influence size and distribution of integrin adhesion complexes. (**a**) Immunofluorescence staining of the IAC component Paxillin (PXN), F-Actin (Phalloidin) and of cell nuclei (Hoechst) was performed. IACs of *ARHGAP29* KD cells appeared larger and more confluent (white arrowheads). Small (nascent) IACs (white arrows) appeared reduced in *ARHGAP29* KD cells. (**b**–**e**) Analysis of IAC morphology confirm increased IAC size and increased appearance of large IACs in *ARHGAP29* KD podocytes. Scatter plot dots indicate mean values of individual cells analyzed (white dots indicate Ctrl. and grey dots KD cells); 3 independent experiments with 25 cells per experiment and genotype (total of 75 cells per genotype) were used for statistical analysis. Percentages indicate relative changes to control for respective IAC size classes. (**f**) Immunofluorescence staining of *ARHGAP29* overexpressing cells show slightly reduced number of large (mature) IACs. (**g**–**j**) Analysis of IAC morphology reveal reduced IAC size and decreased appearance of large IACs. Scatter plot dots indicate mean values of individual cells used for statistical analysis (white dots indicate Ctrl. and grey dots overexpression cells); 4 independent experiments with 25 cells per experiment and genotype (total of 100 cells per genotype) were analyzed. Percentages indicate relative change to control for respective IAC size classes. Error bars indicate mean and S.E.M.; * *p* < 0.05; ** *p* < 0.01; **** *p* < 0.0001; n.s.—not significant.

**Figure 6:**
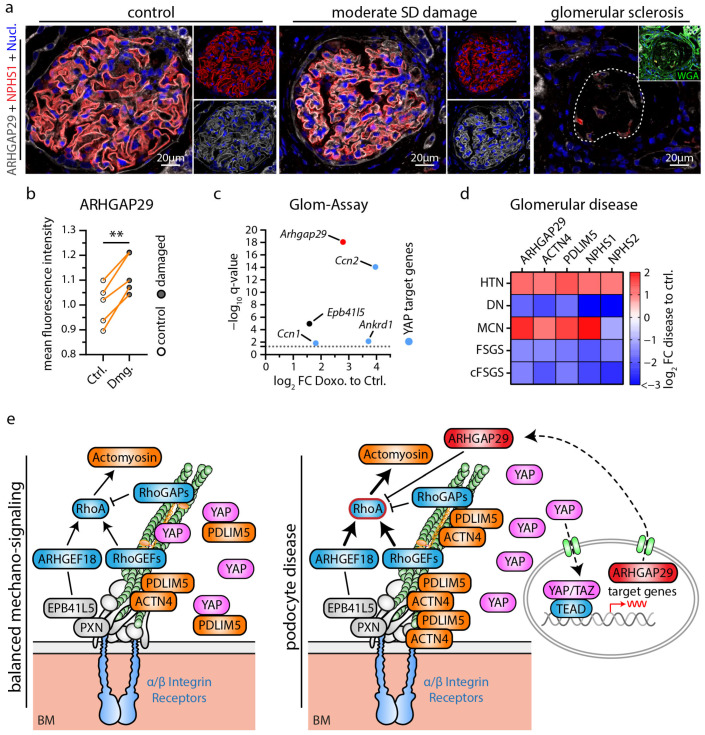
Podocyte damage leads to increased expression of ARHGAP29 counterbalancing RhoA signaling. (**a**) ARHGAP29 immunofluorescence analysis of glomeruli. The slit diaphragm (SD) component NPHS1 was used to label the basolateral podocyte compartment and to indicate glomeruli with damages SD architecture (early and sensitive marker for podocyte damage). Cell nuclei were stained by Hoechst. Representative images show different types of glomerular/podocyte damage within the same kidney sample. Glomeruli were classified as not/very mild damaged (control group), moderate damaged (damaged group) or sclerosed based on the structural integrity of SD (NPHS1) architecture and of overall glomerular structure (evaluated by WGA, NPHS1 and Hoechst staining). Glomerular sclerosis and concomitant podocyte loss causes an overall reduction and loss of ARHGAP29 expression. In contrast, ARHGAP29 expression appears increased in glomeruli with moderate podocyte SD damage. (**b**) Quantification of ARHGAP29 expression (mean fluorescence intensity—MFI) within the podocyte compartment of control (Ctrl.) and moderate damaged (Dmg.) glomeruli (*n* = 5; scatter plot dots indicate mean IF intensity per class and patient used for statistical analysis. Glomerular damage classes were matched per sample for statistical analysis to adjust for global differences in IF signal intensity between individual samples. Orange lines indicate matching classes from one patient; ** *p* < 0.01). (**c**) Isolated murine glomeruli were treated (ex vivo) with Doxorubicin (Doxo) and analyzed for altered gene expression of *Arhgap29*, *Epb41l5* and common YAP target genes. Dotted line indicate adjusted *p*-value (q-value) of 0.05. (**d**) Analysis of mRNA expression levels in glomeruli damaged by different glomerular disease using the Nephroseq database. Heatmap indicates log_2_ fold changes (FC) for selected genes in hypertensive nephropathy (HTN), diabetic nephropathy (DN), minimal change disease (MCD), focal segmental glomerulosclerosis (FSGS) and collapsing FSGS (cFSGS). *ARHGAP29* appears enriched in glomerular diseases with mild damage (HTN, MCD) and reduced in destructive diseases (DN, FSGS, cFSGS). (**e**) Schematic depicting a model for a regulatory function of ARHGAP29 in podocyte disease where increased ARHGAP29 expression counteracts increased mechanosignaling via inhibition of RhoA signaling.

The GraphPad Prism 8 software was used for statistical analysis of experimental data and the preparation of graphs. Scatter plots indicate individual units used for statistical testing (samples, cells, or replicates), as specified in the respective figure legends. Error bars indicate the mean and standard error of the mean (S.E.M.). Statistical tests were applied according to the design and data distribution of each experiment. Unpaired Students *t*-test (Figure 2c,d,f–h, Figure 4f,j,m, Figure 5g–I, Figures S1e, S3c–f and S5g,h), paired Students *t*-test (Figure 6b), unpaired Students *t*-test with Welch’s correction (Figure 3m,n, Figures S3b, S4a,c,d and S5c), one-way ANOVA with Sidak’s multiple comparisons test (Figure 1g, Figure 3j, and Figure 4e,h,l), Brown-Forsythe ANOVA test with Welch ANOVA test and Dunnett’s T3 multiple comparisons test (Figures S1c and S5e,f) or Games-Howell’s multiple comparisons test (Figure 5b,d) were applied. Statistical significance was defined as *p* < 0.05 and significance levels are indicated as * *p* < 0.05, ** *p* < 0.01, *** *p* < 0.001, **** *p* < 0.0001, and non-significant (n.s.) in the respective Figure panels. The number of independent experiments and analyzed units is stated in the figure legends and/or respective methods sections. Scatter plot dots in figures show data (cells, mean value per experimental replicate, patients, etc.) as used for statistical analysis.

## 3. Results

### 3.1. Loss of EPB41L5 Impairs YAP/TAZ-TEAD Complex Mediated Transcriptional Signaling

Previous studies demonstrated that *Epb41l5* is essential for podocyte function and glomerular maturation in mice [17,22,23]. To further confirm the conserved role of *EPB41L5*, we employed the nephrocytes of *D. melanogaster* as an invertebrate model system (Supplemental Figure S1) [41]. RNAi-mediated knockdown (KD) of the *EPB41L5* orthologue Yurt (*Yrt*) revealed a largely reduced uptake of ANF-RFP into pericardial nephrocytes. Further evaluation of *Yrt* KD garland cell nephrocytes by transmission electron microscopy (TEM) demonstrated apical translocation and reduced width of slit diaphragms (SDs), indicating disruption of the filtration barrier as an ultrastructural correlate of the impaired uptake phenotype (Supplemental Figure S1). Given that EPB41L5 appears to be a highly conserved and specific protein for podocyte function, we utilized a previously characterized in vitro model of CRISPR/Cas9-generated human *EPB41L5* wild-type (WT) and knockout (KO) podocytes to identify cell-type-specific disease signatures. Therefore, complementary transcriptome and proteome analyses of respective WT and KO podocytes were performed (Figure 1a) [17,22]. Here, very broad alterations upon deletion of *EPB41L5* were detected within the transcriptional and proteome landscape (Figure 1b,c and Figure S2, Supplementary Tables S1 and S2). Detailed gene set enrichment analysis (GSEA) for GO-Terms demonstrated downregulation of gene sets consisting of extracellular matrix (ECM) and integrin adhesion complex (IAC)-related genes (Supplementary Table S1). These observations further substantiated previous studies on the regulatory function of EPB41L5 on IAC-mediated mechanosignaling and on the composition as well as morphology of the ECM and IACs in podocytes [17,22]. To further delineate regulatory pathways, a detailed transcription factor enrichment analysis (TFEA) was performed (Figure 1d and Supplementary Table S1). Interestingly, this TFEA analysis showed downregulation of genes transcriptionally controlled by YAP/TAZ-TEAD complex-mediated mechanosignaling. Further, detailed workup of transcriptome and proteome datasets demonstrated regulation of a selection of ECM (e.g., *MMP14*, collagens), IACs (e.g., *ITGB3*, *TLN2*, *TNS1*), and the actin cytoskeleton (e.g., *CNN1*, *FCN1*, *FLNA*) components (Supplementary Figure S2 and Supplementary Table S3). Interestingly, some of these genes, similar to collagens, were inversely regulated on transcriptome and protein levels, potentially reflecting the direct involvement of *EPB41L5* in IAC and ECM regulation beyond transcription. To finally test the hypothesis that loss of EPB41L5 is related to diminished YAP/TAZ-TEAD signaling, we mapped commonly used YAP target (indicator) genes and analyzed nuclear localization patterns of YAP/TAZ (Figure 1e–g and Figure S3). Here, we observed diminished nuclear translocation of YAP/TAZ and downregulation of *CTGF*, *CYR61*, *ANKARD1*, and *ARHGAP29* (a recently described YAP/TAZ target gene) [33]. In summary, altered mechanotranduction via the YAP/TAZ pathway and downregulation of TEAD mediated transcription appear to be involved in *EPB41L5*-dependent phenotypes.

### 3.2. Inhibition of YAP/TAZ-TEAD Complex Mediated Transcription Impairs Formation of Cell Protrusions, IACs and Expression of Podocyte Genes

To further dissect YAP/TAZ-TEAD complex-dependent gene transcription in podocytes, we employed a doxycycline-inducible expression system of the YAP/TAZ binding domain of TEAD transcription factors (*TEADi* system) (Figure 2a) [34]. The induction of *TEADi* allows for precisely controlled inhibition of YAP/TAZ-related transcriptional activity and minimizes potential compensatory responses due to this intervention. As the generation of cellular protrusions and tightly controlled adhesion via IACs are essential functions of podocytes, we evaluated parameters such as initial cell spreading as a surrogate parameter for these cellular programs. Here, *TEADi* cells showed delayed initial cell spreading, reduced cell size, a condensed F-Actin cytoskeleton, and altered lamellipodia structure, indicating defective cellular protrusion formation (Figure 2b–d and Figure S4). Moreover, detailed analysis of IAC morphology and structure showed a marked increase in IAC size, based on a relative shift from small (nascent) adhesions towards larger and confluent (mature) adhesions (Figure 2e–i). Interestingly, a comparable phenotype in terms of impaired initial spreading capacity was previously documented in *EPB41L5*-deficient podocytes (also previously shown—Supplementary Figure S3) [22]. On the contrary, loss of EPB41L5 also impacted IAC formation, whereas *TEADi* did not significantly influence these parameters (Figure S3—impaired IAC formation in EPB41L5-deficient podocytes was also previously demonstrated) [17,22]. Given this (partial) phenotypic overlap, we further aimed to identify shared transcriptional signatures via RNA sequencing analysis of *TEADi* cells (Figure 3a). Interestingly, *TEADi* cells showed significant alterations on the transcriptome level, affecting several podocyte-specific genes such as *NPR3*, *AIF1L,* and *RCAN1* (Figure 3b–f and Figure S4, Supplementary Table S4). In a next step, we mapped for shared transcripts downregulated by *TEADi* and by loss of *EPB41L* to identify potential disease-relevant podocyte genes (Figure 3g,h). This mapping strategy resulted in the identification of *ARHGAP29* as a so far not described podocyte gene. These observations were further confirmed by robust downregulation of ARHGAP29 on the protein level as a consequence of either deleting *EPB41L5* or forcing *TEADi* expression. Interestingly, increasing cellular density translated into downregulation of ARHGAP29, mediated via activation of the Hippo—YAP/TAZ signaling pathway (Figure 3i–n).

### 3.3. ARHGAP29 Limits IAC Maturation and Promotes Cell Protrusion Formation

Our extensive transcriptome analysis led to the identification of the RhoGAP ARHGAP29 as a so far uncharacterized YAP target gene in podocytes (Figure 3) [33]. Employing immunofluorescence microscopy demonstrated a pronounced basolateral localization (foot process region) pattern in human glomeruli (Figure 4a). In vitro, ARHGAP29 was detected at the leading edge of lamellipodial cell protrusions and, to a lesser extent, in the cytosolic compartment (Figure 4b). Previous studies described that the RhoGAP function of ARHGAP29 primarily catalyzes the inactivation of active GTP-RhoA into inactive GDP-RhoA [42,43]. No or only minor catalytic activity on RAC1 or CDC42 was reported by these studies. Similar regulatory effects on RhoA signaling activity were observed in podocytes modified by shRNA-mediated knockdown or overexpression of *ARHGAP29* (Figure 4c–f). Given the prominent localization of ARHGAP29 at lamellipodia and the observed *TEADi* phenotypes (Figure 2), further evaluation of cell spreading was performed. Interestingly, knockdown as well as overexpression of *ARHGAP29* impaired initial spreading capacities (30 min after seeding), probably reflecting the requirement for balanced RhoA activity levels for coordinated generation of cellular protrusions (Figure 4g–j). In contrast, completely spread *ARHGAP29* KD cells (24 h after seeding) showed impaired formation and morphology of lamellipodia (stable overexpression cells did not demonstrate a significant alteration of these parameters) (Figure 4k–m and Figure S5). Moreover, KD and overexpression cells showed a condensed and altered F-Actin structure (Figure 4a,f and Figure S5). Given the altered initial cell spreading phenotype, we further analyzed the integrin adhesions of *ARHGAP29* KD cells. Here, *ARHGAP29* KD cells exhibited a shift towards larger and more confluent IACs in line with the IAC phenotype caused by *TEADi* (Figure 5a–e and Figure S5). This shift resulted in an increase in the mean size of individual IACs as well as in the total basal surface area covered by IACs. In contrast, overexpression of *ARHGAP29* led to a minute reduction in mean IAC size and total IAC surface area and to a prominent loss of large (mature) IACs (Figure 5f–j). In summary, *ARHGAP29* expression levels modulate RhoA activity, lamellipodia protrusion, and IAC formation in podocytes.

### 3.4. ARHGAP29 Expression Is Induced as a Consequence of Mild Podocyte Damage

The extensive transcriptome and functional analysis further prompted us to investigate the potential role of ARHGAP29 in glomerular disease. Here, we analyzed localization patterns of ARHGAP29 in mildly damaged glomeruli in the context of chronic kidney disease with arterial hypertension and observed increased levels of ARHGAP29 in minimally damaged glomeruli (Figure 6a,b). Selected glomeruli showed disturbed localization patterns of the SD marker Nephrin (a highly sensitive marker for reduced SD density and foot process widening), but at the same time exhibited no manifest signs of overt glomerular sclerosis. Importantly, no obvious signs of mislocalization of ARHGAP29 were observed. In contrast, sclerosed areas of glomeruli exhibited a strong reduction in ARHGAP29 expression, probably related to podocyte loss and dedifferentiation. Moreover, analysis of injured (ex vivo) murine glomeruli (Doxorubicin induced podocytopathy) revealed transcriptional upregulation of *Arhgap29* and further YAP target genes (*Ccn1*, *Ccn2*, and *Ankard1*) (Figure 6c). These findings were further substantiated by the analysis of human glomerulopathy using the Nephroseq database (Figure 6d and Supplementary Table S5). In these data sets, *ARHGAP29* mRNA expression appeared enriched in glomerular diseases usually presenting with milder damage patterns such as hypertensive nephropathy (HTN) and minimal change disease (MCD). In contrast, destructive glomerular diseases such as diabetic nephropathy (DN), focal segmental glomerulosclerosis (FSGS), and collapsing FSGS (cFSGS) showed reduced levels of *ARHGAP29* expression. Interestingly, similar expression patterns were detected for *ACTN4* and *PDLIM5*, two IAC and actin cytoskeleton-associated scaffold proteins involved in the regulation of YAP/TAZ signaling [44]. Notably, IAC localization of ACTN4 and PDLIM5 is promoted by EPB41L5-dependent mechano- and RhoA/ROCK/Myosin-II signaling [22]. Based on these observations, we propose a model for a potential protective function of ARHGAP29 to maintain the glomerular filtration barrier in podocyte disease (Figure 6e). In conditions of increased mechanosignaling (activated YAP/TAZ signaling, e.g., via EPB41L5, PDLIM5, ACTN4), this might translate into upregulation of ARHGAP29 to counterbalance mechanosignaling via inhibition of RhoA signaling.

## 4. Discussion

Based on previous studies demonstrating the essential role of EPB41L5 for podocyte function, we employed established CRISPR/Cas9-mediated knockout lines as a model tool to identify relevant disease signatures (Figure 1). Thereby, we identified reduced activity of the YAP/TAZ-TEAD transcription factor complex, partially explaining the observed transcriptome alterations. These findings are further supported by previous studies reporting a role for EPB41L5 in podocyte mechanotransduction via modulation of IACs and RhoA-actomyosin signaling [17,21,22]. Interestingly, loss of EPB41L5 impairs the recruitment of ACTN4 and PDLIM5 towards IACs, while this recruitment has been shown to activate YAP/TAZ signaling [22,44]. Hippo-YAP/TAZ signaling is a well-established and central pathway influencing podocyte function in physiology and disease [29,30,31,45]. However, the complex regulation and related transcriptional consequences in the context of podocytes have so far only been characterized to a limited extent. While previous studies mainly focused on YAP/TAZ or further upstream regulatory elements, we chose a different strategy and aimed to examine the direct consequences of precisely altered transcriptional regulation of the YAP/TAZ-TEAD complex by using the *TEADi* system [34]. Subsequent transcriptome analysis revealed significant transcriptional changes in several genes with podocyte-specific expression patterns, including reduced expression of *ARHGAP29* (Figure 3).

ARHGAP29 was previously detected as a potential component of an IAC subset and as a proximity interactor of RAC1 by proteomic analysis of these compartments [46,47]. However, the cellular function of ARHGAP29 in podocytes has not been described so far. ARHGAP29 is a RhoGAP for RhoA and a recently verified target gene of the YAP/TAZ-TEAD transcription factor complex (Figure 4) [33,42,43]. Despite ARHGAP29 being mainly considered a RhoGAP for RhoA in the literature, we cannot exclude direct or secondary effects on RAC1 or CDC42 activity. Notably, activation of RhoA is a driving factor of podocyte disease [27,48], and the RhoA-actomyosin signaling pathway promotes Hippo-YAP/TAZ signaling [49]. Therefore, increased ARHGAP29 expression probably represents a negative feedback loop for the RhoA–YAP/TAZ pathway [33,50]. On the contrary, loss of *EPB41L5* reduces RhoA activity, which in turn leads to reduced *ARHGAP29* expression (Figure 1 and Figure 3) [17]. In the context of impaired RhoA activation, reduction of ARHGAP29 activity might exert a counterbalancing effect on RhoA inactivation-related phenotypes such as impaired IAC maturation or cellular protrusion generation.

Our localization studies demonstrated ARHGAP29 at the foot processes of podocytes and the protrusion edge (leading edge) of lamellipodia (Figure 4). Knockdown of *ARHGAP29* impaired initial cell spreading, F-Actin structure and affected the morphology of the leading edge (Figure 4). In line with this, similar spreading defects were also observed in *TEADi* and *EPB41L5* knockout podocytes (Figure 2 and Figure S3) [17]. Previous studies on ARHGAP29 described similar localization patterns and lamellipodia protrusion defects in endothelial cells and YAP/TAZ mediated F-Actin alterations, supporting our observations in the podocyte context [33,51,52]. Mechanistically, precise spatio-temporal regulation of RhoA activity at the leading edge is essential during lamellipodia based protrusion [53,54,55]. In this context, ARHGAP29 is probably required for the coordinated inactivation of RhoA during leading edge formation. Effective and coordinated fine-tuning of small RhoGTPase signaling is also essentially required at the IAC compartment [43,56]. Indeed, IAC analysis of *TEADi* expressing and *ARHGAP29* knockdown cells demonstrated a significant shift of individual IAC sizes towards larger and confluent IACs (Figure 2 and Figure 5). This phenotype is an expected result of elevated RhoA—actomyosin signaling on IACs and has been observed as a consequence of YAP/TAZ knockdown in endothelial cells [56,57,58]. Under physiological conditions, RhoGTPase regulation at IACs is mediated by IAC associated RhoGEFs & RhoGAPs such as ARHGEF7 (β-Pix) or SRGAP1 to control IAC maturation [8,57]. However, others and we did not detect any obvious localization of ARHGAP29 to mature IACs (Figure 4) [43]. Lamellipodia based cell protrusion, retrograde actin flow, IAC turnover and RhoGTPase—actomyosin signaling are reciprocally interlinked processes [56,59]. For these reasons, defective lamellipodia protrusion and subsequently impaired retrograde actin flow, IAC turnover and RhoGTPase signaling represent an alternative explanation of this IAC phenotype, not requiring direct action of ARHGAP29 on IACs. Supporting this hypothesis, ablation of lamellipodia in podocytes and other cell types by deletion of the Arp2/3 complex causes a very similar IAC phenotype characterized by a shift from smaller (nascent) to enlarged (mature) IACs [60,61].

Reorganization of the actin cytoskeleton and of cell-matrix adhesions towards a contractile and mechanically enforced RhoA-dependent actomyosin cytoskeleton is a common pattern in podocytopathy and considered a driving factor of glomerular disease [9,12,19,62]. Analysis in human kidney tissue showed increased *ARHGAP29* expression in early glomerular disease stages (Figure 6). In line with the above proposed regulatory feedback loop, ARHGAP29 might exert a protective effect in podocyte disease by limiting IAC and actin cytoskeleton remodeling and promoting cell protrusion formation. Future studies will have to investigate the role of *ARHGAP29* in in vivo models of glomerular disease and its potential for targeted therapeutic approaches.

## Data Availability

The data presented in this study are available in the article or Supplementary Materials. Sequencing data have been deposited in NCBI Gene Expression Omnibus and are accessible via GEO series, accession numbers GSE220299 and GSE220221. The LC-MS/MS data was uploaded to the MassIVE repository (part of the ProteomeXchange consortium). Data can be accessed using the MassIVE accession number MSV000091196.

## Supplementary Materials

The following supporting information can be downloaded at: https://www.mdpi.com/article/10.3390/cells12131795/s1, Figure S1: Analysis of Yurt (*Yrt*) knockdown in *Drosophila melanogaster* nephrocytes; Figure S2: Transciptome and proteome analysis of *EPB41L5* KO podocytes; Figure S3: Analysis of *EPB41L5* KO podocytes; Figure S4: Analysis of *TEADi* KO podocytes; Figure S5: Analysis of *shARHGAP29* podocytes; Figure S6: Analysis of human glomeruli; Table S1: Transciptome, GSEA and TFEA analysis of *EPB41L5* KO podocytes; Table S2: Proteome analysis of *EPB41L5* KO podocytes; Table S3: Comparative analysis of transciptome and proteome datasets of *EPB41L5* KO podocytes; Table S4: Transciptome, GSEA, TFEA and comparative analysis of *TEADi* podocytes; Table S5: Nephroseq analysis of *ARHGAP29* in glomerular disease datasets.
