# Supplementary material for: A YAP/TAZ–ARHGAP29–RhoA Signaling Axis Regulates Podocyte Protrusions and Integrin Adhesions
Source: Cells. 2023 Jul 6;12(13):1795. doi: 10.3390/cells12131795 (PMC10340513; doi:10.3390/cells12131795)
Supplement: Supplementary file 1 [file cells-12-01795-s001.zip › Supplementary Figures.pdf]

Supplementary Figures

Supplemental Figure S1

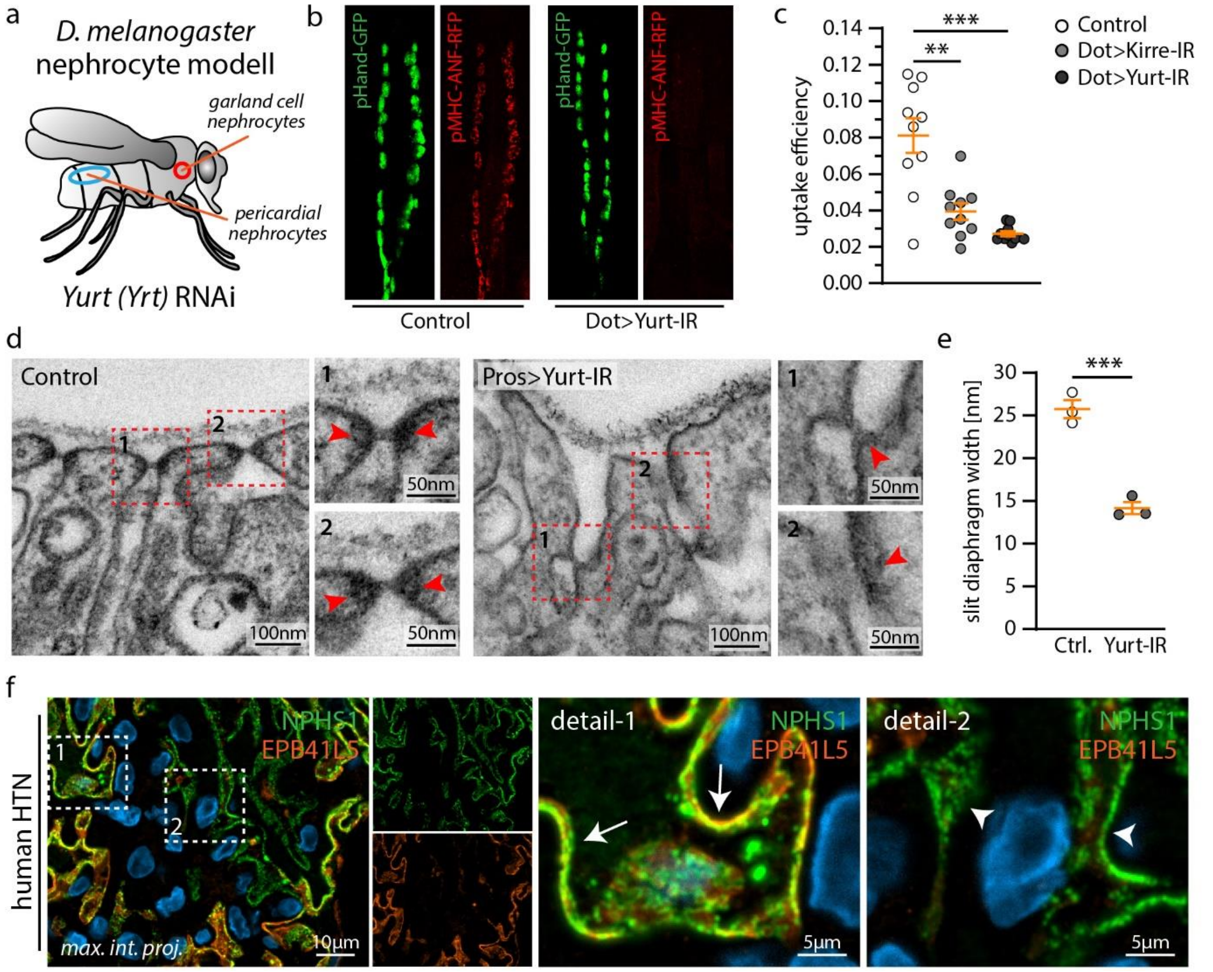

(a–e) RNAi knockdown (KD) of the *EPB41L5* orthologue Yurt (*Yrt*) in *Drosophila* melanogaster garland cell nephrocytes using Pros-Gal4 and Dot-Gal4 to target pericardial nephrocytes. (b&c) ANF-RFP uptake assay revealed impaired ANF-RFP (red) uptake in *Yrt* KD nephrocytes. Nephrocytes were labeled by Hand-GFP (green); 10 biological replicates per genotype were analyzed. Knockdown of the slit diaphragm (SD) component *Kirre* was analyzed as positive control. (d&e) Transmission electron microscopy reveals apical translocation and reduced width of SDs as a consequence of *Yrt* KD (red arrowheads indicate SDs; at least 200 slit membranes averaged from 3 different biological replicates per genotype were measured). (f) Immunofluorescence analysis of EPB41L5 in human glomerular disease (HTN – hypertensive nephropathy). The SD component Nephrin (NPHS1) was used as marker for the basal podocyte compartment and to indicate regions of disrupted SD architecture. White arrows indicate regions with increased or stable EPB41L5 localization and white arrowheads indicate regions with reduced or lost EPB41L5 localization (maximum intensity projection of z–stack images are shown). Scatter plots show individual animals analyzed; scatter plot dots show data used for statistical analysis; error bars indicate mean and S.E.M.; **p < 0.01; ***p < 0.001.

Supplemental Figure S2

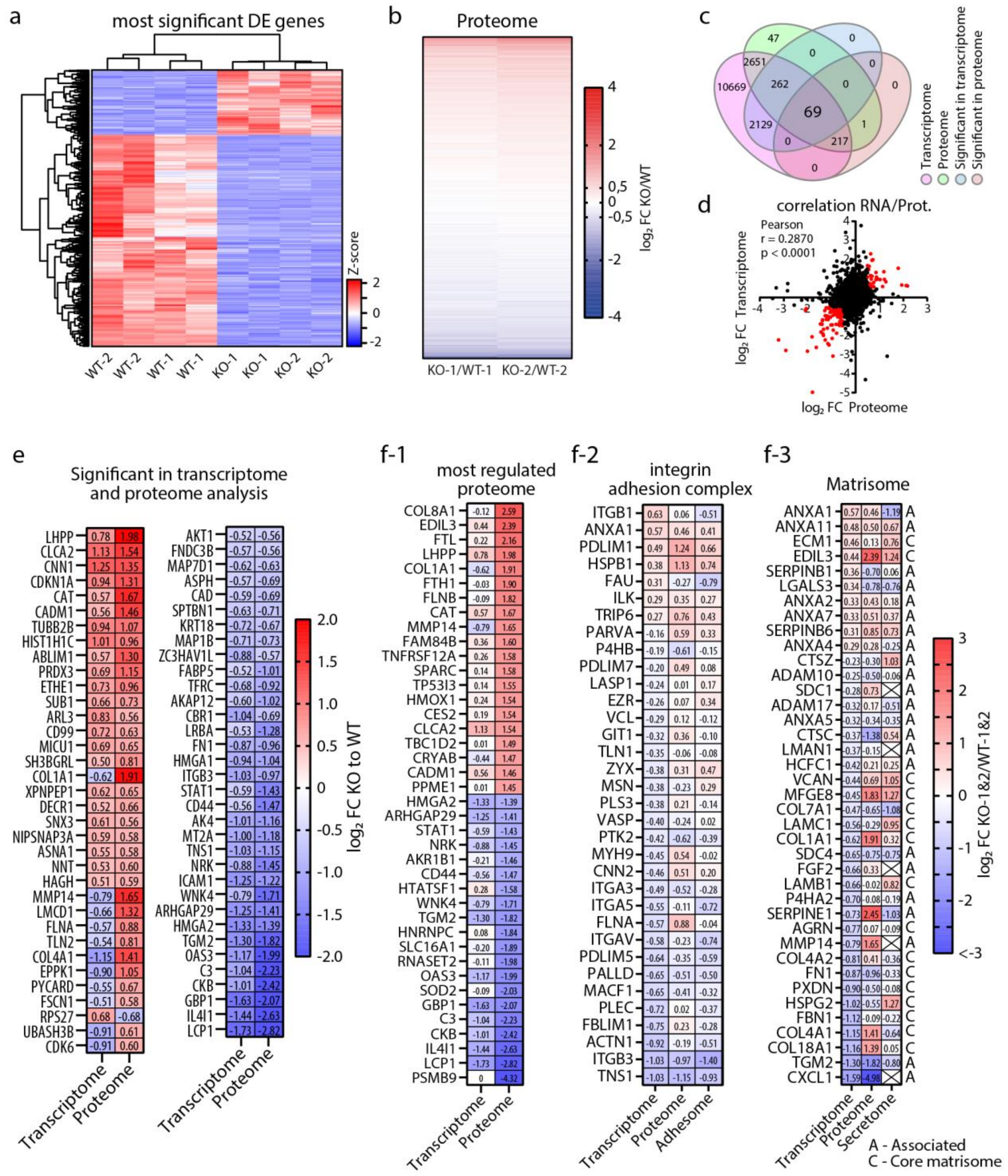

(a) Heatmap shows Z-scores for most significant regulated gene transcripts by knockout of *EPB41L5* (log_2 fold change >1 or <-1 and adjusted. p-value <0.05). (b) Heatmap shows relative regulation of proteins detected by SILAC based proteomics of *EPB41L5* WT and KO podocytes. Only proteins with assigned log_2 fold changes (FC) in both comparisons are shown. (c) Venn diagram analysis indicates significant regulation of 69 genes on transcript and proteins level in *EPB41L5* KO podocytes. Significance in transcriptome analysis was defined as log_2 fold change >0.5 or <-0.5 and adjusted p-value <0.05. Significance in proteome analysis was defined as log_2 fold change >0.5 or <-0.5 in both sample pairs. (d) Correlation analysis of these proteins with transcriptome analysis demonstrate partial and significant correlation of transcriptome and proteome datasets. Pearson correlation coefficient was calculated as indicated. Scatter plot dots indicate individual transcripts/proteins.

Transcripts/proteins with correlating log_2 fold changes (FC) >0.5 or <-0.5 in both datasets are labeled in red. (e) Heatmap shows log_2 fold changes (FC) for these 69 transcripts and proteins. (f-1) Heatmap shows the 20 most up and 20 most down regulated proteins by proteomics analysis of *EPB41L5* KO podocytes. (f-2) Heatmap shows integrin adhesion complex transcripts/proteins that are significant regulated by transcriptome analysis (adjusted p-value >0.05) and detected in whole cell proteome and adhesome analysis of *EPB41L5* KO podocytes (adhesome – proteome analysis of integrin adhesion complexes was published before) [1]. (f-3) Heatmap shows matrisome transcripts/ proteins that are significant regulated by transcriptome analysis (adjusted p-value >0.05) and detected in whole cell proteome analysis of *EPB41L5* KO podocytes. Corresponding secretome analysis is shown (secretome – proteome analysis of secreted proteins was published before) [2].

Supplemental Figure S3

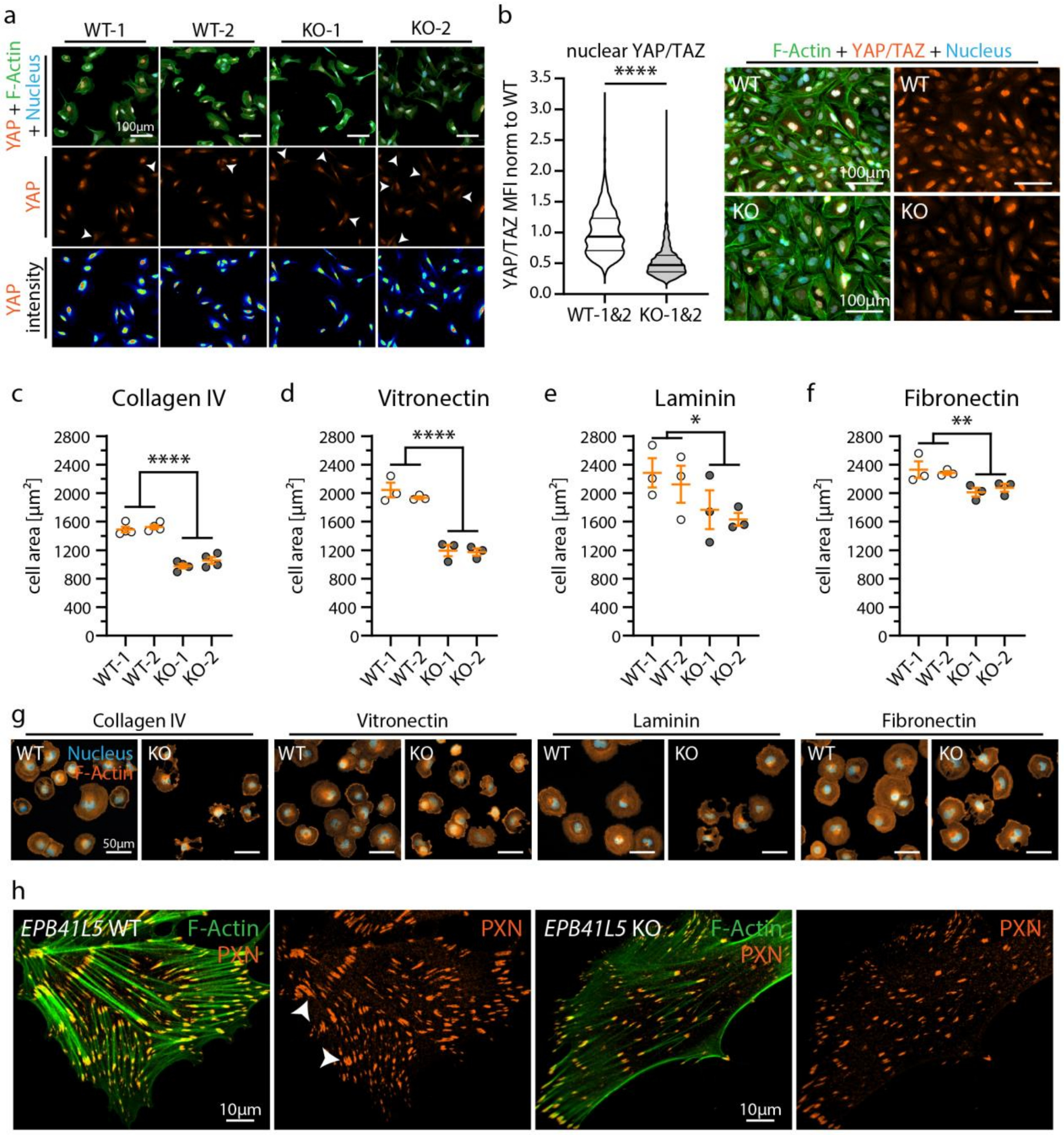

(a) Overview images corresponding to main Figure 1. Immunofluorescence analysis of nuclear translocation of YAP confirms reduced YAP levels in *EPB41L5* KO podocytes. Rainbow color images

display the distribution of YAP fluorescence intensity. Cells were co-stained by Phalloidin (F-Actin) and Hoechst (cell nucleus). White arrowheads indicate cell nuclei with low nuclear YAP intensity. (b) Immunofluorescence analysis of nuclear translocation of YAP/TAZ reveals reduced YAP/TAZ levels in *EPB41L5* KO podocytes. Cells were cultured for 3 days to sub-confluence before staining for Phalloidin (F-Actin) and Hoechst (cell nucleus). Violin blots indicate distribution of mean nuclear fluorescence intensities (MFIs) of individual cells of 2 experiments analyzed; cells of individual WT and KO cells lines were pooled for analysis (over 800 cells per condition were analyzed). Bars indicate median and quartiles; ****p < 0.0001. (c-g) Cell spreading analysis of *EPB41L5* WT and KO podocytes shows impaired cell spreading on collagen IV, vitronectin, laminin or fibronectin coated glass coverslips. Cells were stained by Phalloidin (F-Actin) and Hoechst (cell nucleus). Scatter plot dots indicate mean cell size of 3 or 4 experiments analyzed. Values of WT-1 & -2 and KO-1 & -2 were pooled for statistical analysis. Error bars indicate mean and SEM; scatter plot dots show data used for statistical analysis; *p < 0.05; **p < 0.01; ****p < 0.0001. (h) Representative immunofluorescence images of podocyte stained for the IAC component PXN, F-Actin (Phalloidin) and of cell nuclei (Hoechst) demonstrates reduced number and size of IACs in *EPB41L5* KO podocytes, as previously reported [1, 2].

Supplemental Figure S4

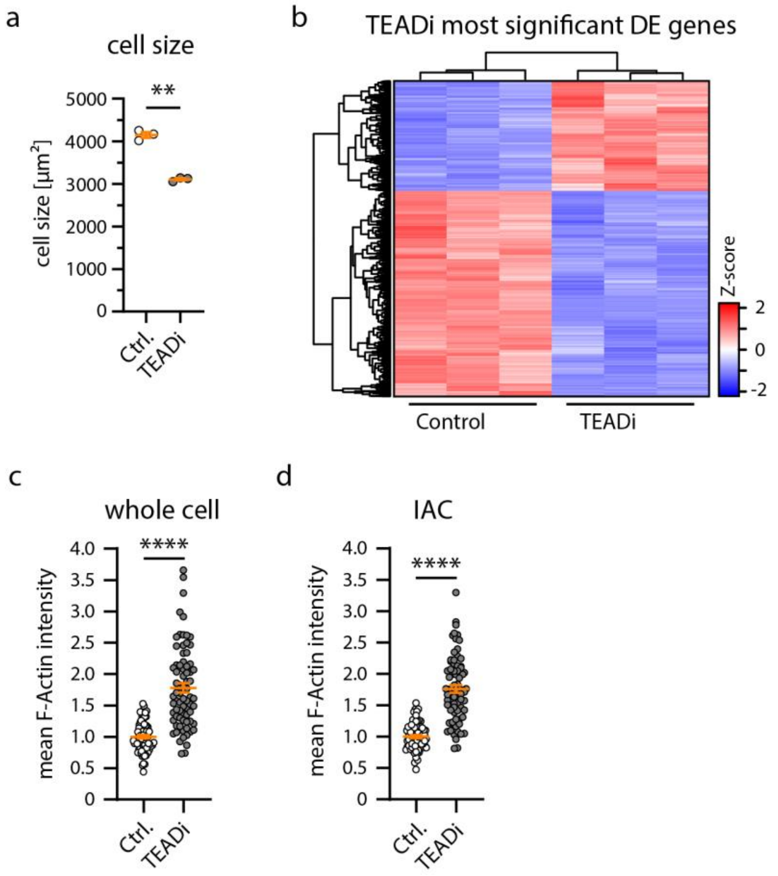

(a) Cell size is reduced in *TEADi* podocytes 24 hours after seeding. Scatter plot dots indicate mean cell size of 3 experiments analyzed. (b) Heatmap shows Z-scores for most significant regulated gene transcripts by *TEADi* expression (log_2 fold change >0.4 or <-0.4 and adjusted p-value <0.0001). (c&d) Phalloidin (F-Actin) mean fluorescence intensity was measured in the whole cell and integrin adhesion complex (IAC) compartment. Scatter plot dots indicate individual cells analyzed (3 experiments and 25 cells per experiment and genotype were analyzed). Error bars indicate mean and SEM; scatter plot dots show data used for statistical analysis; **p < 0.01; ****p < 0.01.

Supplemental Figure S5

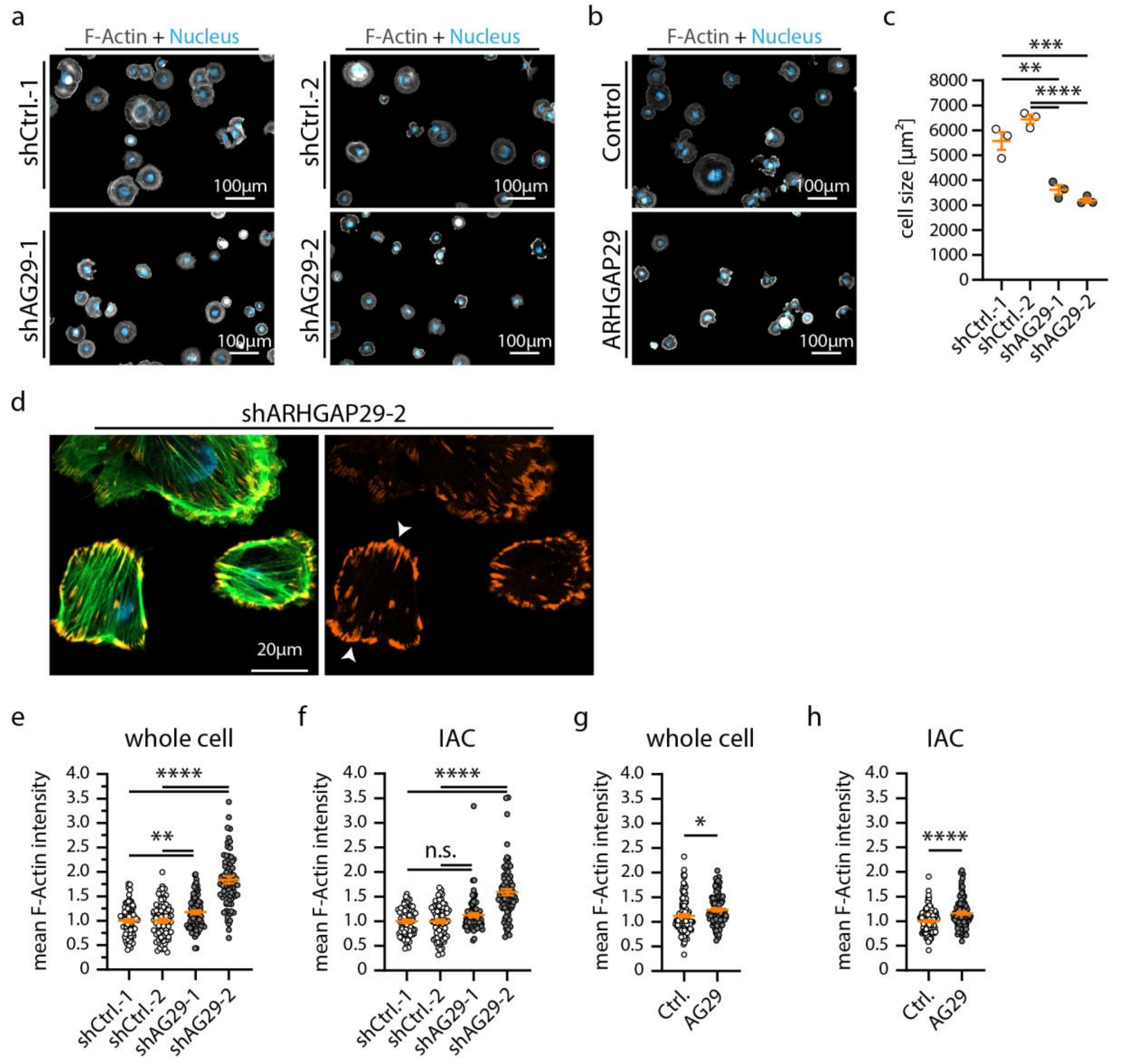

(a&b) Overview images corresponding to cell spreading analysis presented in main figure 4 (cells were stained by Phalloidin (F-Actin) and Hoechst (cell nucleus)). (c) Analysis of cell size of fully spread cells (24 hours) on collagen IV. *ARHGAP29* KD cells exhibited reduced cell areas. Scatter plot dots indicate mean cell size of 3 experiments analyzed. Error bars indicate mean and SEM; scatter plot dots show data used for statistical analysis; **p < 0.01; ***p < 0.001; ****p < 0.0001. (d) Immunofluorescence analysis of *ARHGAP29* shRNA-2 corresponding to main figure 5. Immunofluorescence staining of the IAC component Paxillin (PXN), F-Actin (Phalloidin) and of cell nuclei (Hoechst) was performed. (c-h) Phalloidin (F-Actin) mean fluorescence intensity was measured in the whole cell and integrin adhesion complex (IAC) compartment. Scatter plot dots indicate individual cells analyzed (3 experiments (e&f) or 4 experiments (g&h) and 25 cells per experiment and genotype were analyzed). Error bars indicate mean and SEM; scatter plot dots show data used for statistical analysis; n.s. – not significant; *p < 0.05, **p < 0.01; ****p < 0.01.

Supplemental Figure S6

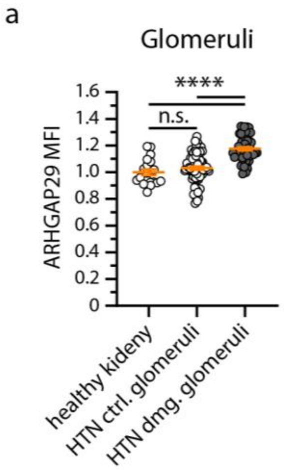

(a) Additional data to main Figure 6a&b. Quantification of ARHGAP29 expression (mean fluorescence intensity - MFI) within the podocyte compartment. Scatter plot dots indicate pooled glomeruli of 5 patients with hypertensive nephropathy (HTN) classified as moderate damaged (dmg.) (49 glomeruli) or not/very mild damaged (control group) (78 glomeruli). Analysis of one healthy kidney (20 glomeruli) was included as reference. Error bars indicate mean and SEM; scatter plot dots show data used for statistical analysis; n.s. – not significant; ****$p < 0.0001$.

References

1. Schell, C. *et al.* The FERM protein EPB41L5 regulates actomyosin contractility and focal adhesion formation to maintain the kidney filtration barrier. *Proc Natl Acad Sci U S A* **114**, E4621-E4630 (2017).
2. Maier, J.I. *et al.* EPB41L5 controls podocyte extracellular matrix assembly by adhesome-dependent force transmission. *Cell Rep* **34**, 108883 (2021).
